# Ultra-thin g-C_3_N_4_/MFM-300(Fe) heterojunctions for photocatalytic aerobic oxidation of benzylic carbon centers†

**DOI:** 10.1039/d1ma00266j

**Published:** 2021-07-05

**Authors:** Chengcheng Liu, Tian Luo, Alena M. Sheveleva, Xue Han, Xinchen Kang, Sergei Sapchenko, Floriana Tuna, Eric J. L. McInnes, Buxing Han, Sihai Yang, Martin Schröder

**Affiliations:** Department of Chemistry, University of Manchester, Oxford Road Manchester M13 9PL UK sihai.yang@manchester.ac.uk m.schroder@manchester.ac.uk; Institute of Molecular Sciences and Engineering, Institute of Frontier and Interdisciplinary Science, Shandong University Qingdao 266237 China; Photon Science Institute, University of Manchester Oxford Road Manchester M13 9PL UK; Beijing National Laboratory for Molecular Sciences, CAS Key Laboratory of Colloid, Interface and Chemical Thermodynamics, Institute of Chemistry, Chinese Academy of Science Beijing 100190 China

## Abstract

*In situ* growth of the metal–organic framework material MFM-300(Fe) on an ultra-thin sheet of graphitic carbon nitride (g-C_3_N_4_) has been achieved *via* exfoliation of bulk carbon nitride using supercritical CO_2_. The resultant hybrid structure, CNNS/MFM-300(Fe), comprising carbon nitride nanosheets (CNNS) and MFM-300(Fe), shows excellent performance towards photocatalytic aerobic oxidation of benzylic C–H groups at room temperature under visible light. The catalytic activity is significantly improved compared to the parent g-C_3_N_4_, MFM-300(Fe) or physical mixtures of both. This facile strategy for preparing heterojunction photocatalysts demonstrates a green pathway for the efficient and economic oxidation of benzylic carbons to produce fine chemicals.

## Abstract 2

*In situ* growth of the MFM-300(Fe) on an ultra-thin sheet of graphitic carbon nitride, formed by exfoliation using supercritical CO_2_, is reported. The resultant composite CNNS/MFM-300(Fe) shows excellent performance in photocatalytic aerobic oxidation of benzylic C–H groups.

## Graphical abstract

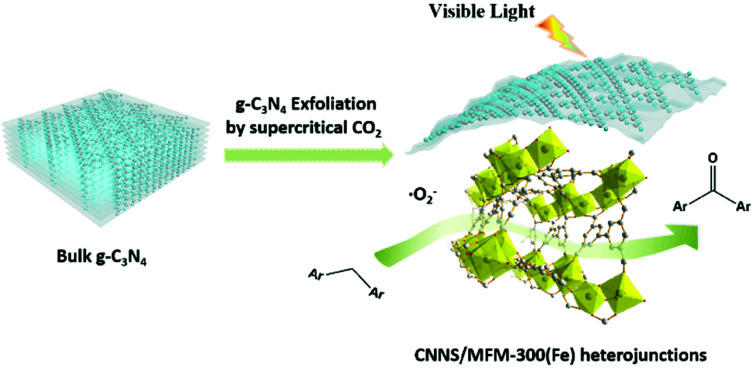

## Introduction

Photocatalysis holds increasing promise to tackle resource depletion by utilising sunlight as the primary energy source.^1–4^ While TiO_2_ and many other semiconductors are studied widely as photocatalysts for the degradation of organic pollutants and the synthesis of chemicals using solar energy, these materials generally only adsorb in the UV region.^4,5^ Great efforts have been devoted to developing photocatalysts with strong absorbance in the visible region, and precious metal (*e.g.*, ruthenium and iridium) complexes and organic dyes have been developed as state-of-the-art systems.^1,6,7^ Metal–organic framework materials (MOF) have been investigated as photocatalysts for water splitting, CO_2_ reduction and organic transformations.^8–10^ Their high porosity and potential active sites can facilitate catalysis and the transport of substrates and products.^11,12^ However, the wide band gap of MOFs greatly limits their adsorption of visible light. One promising solution is to incorporate MOFs with a photosensitizer that absorbs visible light, such as semiconductors of narrow band gap, to form a heterojunction photocatalyst. This provides an internal electric field between the host catalyst and co-catalyst to achieve electron–hole pair separation and induce improved carrier migration.^13^ Here, graphitic carbon nitride (g-C_3_N_4_) has been chosen as the sensitiser due to its desirable electronic and optical properties, high chemical stability, and low-cost of production.^14–16^ However, bulk g-C_3_N_4_ has a low surface area and suffers from fast recombination of the photo-generated electron–hole pairs. Two-dimensional carbon nitride nanosheets (CNNS), prepared by exfoliation of bulk g-C_3_N_4_, promotes high photocatalytic activity by virtue of its increased band gap, enhanced electron-transport and prolonged lifetime.^14^ Sonication-assisted exfoliation often uses large amounts of high boiling point toxic and expensive solvents (*e.g.* 5,5-dimethyl-1-pyrroline *N*-oxide, 1-methyl-2-pyrrolidinone and *N*-vinylpyrrolidone).^17^ In contrast, supercritical CO_2_ (scCO_2_) is environmentally benign and can be readily removed by depressurisation after exfoliation.^18^

Herein, we report the first example of scCO_2_-assisted exfoliation of bulk g-C_3_N_4_ to give CNNS of ∼3 nm thickness. These ultra-thin nanosheets show strong absorbance of visible light and can act as substrates for the *in situ* growth of nano-scale MOFs with a crystallite width of 300 nm in a one-pot synthesis. In this method, scCO_2_ plays a critical role in the exfoliation of g-C_3_N_4_, which is then used to generate nano-sized MFM-300(Fe) and its uniform dispersion on CNNS. The resultant heterojunctions, CNNS/MFM-300(Fe), are shown to have exceptional catalytic activity and stability for the aerobic photo-oxidation of benzylic carbons using visible light (400–1100 nm). The photocatalytic aerobic oxidation of benzylic carbon driven by visible light is important but challenging, and the activation of O_2_ is critical in this process.^19–21^ Here, electron paramagnetic resonance (EPR) spectroscopy was employed to study the formation of superoxide anion radicals ^•^O_2_^−^ obtained by one-electron reduction of O_2_ with the photogenerated electrons transferred from the CNNS/MFM-300(Fe) composite.

## Results and discussion

### Synthetic procedures, and characerizations of CNNS/MFM-300(Fe).

**Scheme 1:**
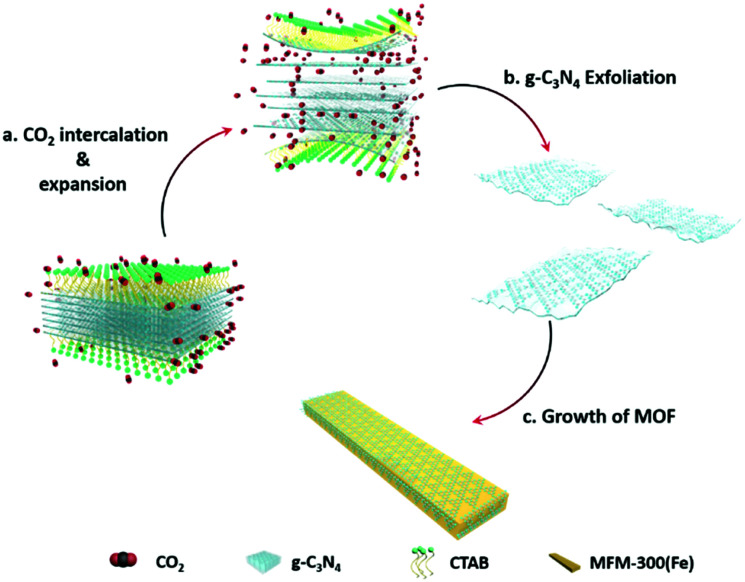
Schematic illustration of scCO_2_-assisted exfoliation of bulk g-C_3_N_4_ and the *in situ* synthesis of the CNNS/MFM-300(Fe) composite.

**Fig. 1:**
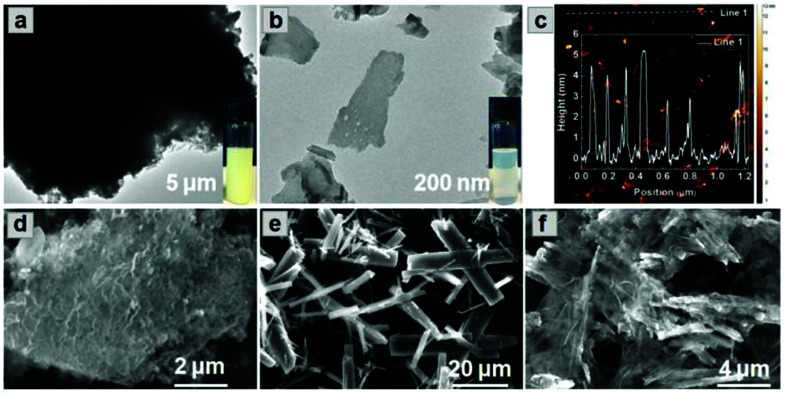
TEM images of (a) bulk g-C_3_N_4_ and (b) CNNS exfoliated using scCO_2_/CTAB. (c) AFM image of CNNS, inset: height of the CNNS as determined by AFM; SEM images of (d) bulk g-C_3_N_4_, (e) MFM-300(Fe), and (f) CNNS/MFM-300(Fe) composite. The composites shown here all have a g-C_3_N_4_ : MOF wt : wt ratio of 7 : 3.

Combining the diffusion of a gas and solvation of a liquid, scCO_2_ can readily insert between layers of bulk g-C_3_N_4_, thus expanding the distance and decreasing the interaction between adjacent layers (Scheme 1a). The addition of the surfactant hexadecyl trimethyl ammonium bromide (CTAB) further assists the curvature and delamination of the ultra-thin nanosheets by forming micelles on the layers (Scheme 1b). Transmission electron microscopy (TEM) of bulk g-C_3_N_4_ and of exfoliated nanosheets clearly shows the transparency of CNNS (Fig. 1a and b). The thickness of the exfoliated CNNS was measured by atomic force microscopy (AFM) to be within the range 0.5–5.5 nm for 45 selected flakes of CNNS (Fig. 1c). The obtained CNNS were further characterised by powder X-ray diffraction (PXRD), Fourier-transform infrared spectroscopy (FT-IR) and thermogravimetric analysis (TGA), and these all show distinct features compared with bulk g-C_3_N_4_ thus demonstrating the successful exfoliation process (Fig. S1, ESI†). UV-vis absorption spectroscopy and Mott–Schottky (MS) analysis confirm the enhanced photoelectric properties of exfoliated CNNS (Fig. S2, ESI†).^22,23^ The carrier density of CNNS is 5.9 times that of bulk g-C_3_N_4_, which can greatly facilitate charge transport within a potential photocatalytic process.^24^

MFM-300(Fe)^25^ was chosen to prepare heterojunction photo-catalysts due to its structural robustness and stability and the high environmental abundance of Fe-based salts. The structure of MFM-300(Fe) consists of helical chains of [FeO_4_(OH)_2_] moieties bridged by 4,4′-bipyridyl-3,3′,5,5′-tetracarboxylate (L^4−^) to generate a porous framework with square-shaped channels. This affords control of product selectivity *via* regulation of the accessibility of reactants and products to and within the pores based upon their size and shape.^25,26^ The CNNS/MFM-300(Fe) heterojunctions were prepared *via* a one-pot synthesis. FeCl_3_, H_4_L, bulk g-C_3_N_4_ and CTAB were mixed in DMF in an autoclave at 393 K for 1 day under compressed CO_2_ to promote the simultaneous exfoliation of g-C_3_N_4_ and crystallisation of MFM-300(Fe) (Scheme 1c). CNNS/MFM-300(Fe) was obtained as yellow microcrystalline powders. The CNNS : MOF ratio ranged from 9 : 1 to 3 : 7, and retention of the crystal structure of MFM-300(Fe) within CNNS/MFM-300(Fe) was confirmed by PXRD (Fig. S3 and S4, ESI†). FTIR spectra (Fig. S5, ESI†) show that the stretching modes of the aromatic CN heterocycles in the composite are blue-shifted to 1243, 1316 and 1567 cm^−1^ compared with bulk g-C_3_N_4_,^27,28^ and those of the MOF in the composite are consistent with pristine MFM-300(Fe). The optical response of the composite was investigated by diffuse-reflectance UV-vis spectroscopy (Fig. S6, ESI†). CNNS/MFM-300(Fe) exhibits an improved absorption of visible light compared to MFM-300(Fe) and g-C_3_N_4_, and SEM confirms that the g-C_3_N_4_ multilayers have exfoliated into nanosheets, which combine with nanorods of MFM-300(Fe) to form a composite (Fig. 1d–f). The elemental mapping of the composite shows uniform distribution of MFM-300(Fe) across the CNNS (Fig. S7, ESI†). The particle size of MFM-300(Fe) (0.3 × 3 μm) in the composite is much smaller than that of the pristine MOF (10 × 40 μm), leading potentially to exposure of additional active sites and reducing the recombination rate of the photogenerated charge carriers.^29^

### Photocatalytic activity test

**Fig. 2:**
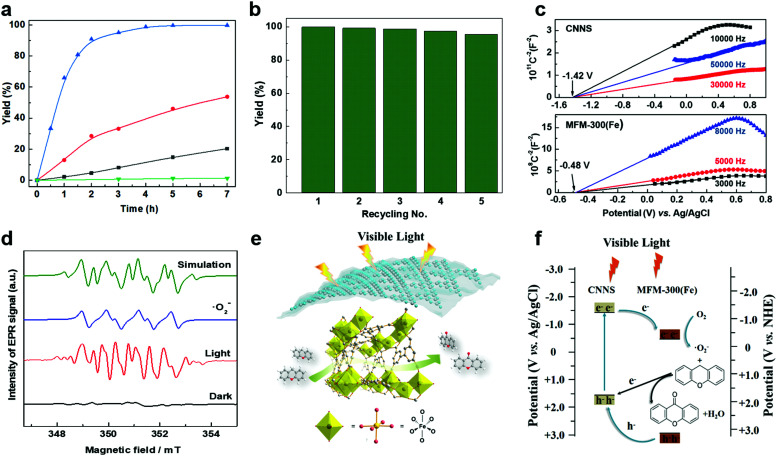
(a) Formation of xanthone catalysed by MFM-300(Fe) (black), g-C_3_N_4_ (red), CNNS/MFM-300(Fe) composites (blue) and thermal catalysis with CNNS/MFM-300(Fe) composites at 343 K in the dark (green). (b) Catalytic performance over five continuous cycles of oxidation of xanthene. (c) Mott–Schottky plots for CNNS (top) and MFM-300(Fe) (bottom) at different frequencies. (d) EPR spectra spin-trapping with DMPO of CNNs/MFM-300(Fe) under dark conditions (black) and after 10 min of visible light irradiation (red). Signals for ˙O_2_^−^ (blue) and simulated (green) of the spectrum after irradiation. (e) Schematic diagram for the photocatalytic process. (f) Schematic illustration of charge separation of CNNS and MFM-300(Fe) and charge-transfer between the bands of CNNS/MFM-300(Fe) heterojunctions. The CNNS/MFM-300(Fe) composites shown here contain g-C_3_N_4_ : MOF in a wt : wt ratio of 7 : 3.

Oxidation reactions using hydroperoxides as strong oxidising agents at elevated temperatures are widely used in the industrial synthesis of fine chemicals.^30,31^ A highly desirable alternative is to use molecular O_2_ as a green oxidant, visible-light as the energy source, and an inexpensive and readily recoverable photocatalyst(s) to drive the oxidation reaction at room temperature. For photocatalytic aerobic oxidation of benzylic carbon centres, homogeneous catalysts (*e.g.* Mn^III^ corrolazine complex,^19^ 2,3-dichloro-5,6-dicyano-1,4-benzoquinone^32^) with co-catalysts and heterogeneous catalysts (*e.g.* g-C_3_N_4_, CdS^20^) have been tested. In this work, we report such a process using 1 bar O_2_ and CNNS/MFM-300(Fe) for the photocatalytic oxidation of xanthene using visible light at room temperature (Fig. 2a). Toluene is the most suitable solvent compared with MeCN and trifluorotoluene for this atom efficient process for a range of substrates (Table 1). A CNNS/MFM-300(Fe) composite with a g-C_3_N_4_ content of 70 wt% shows the best catalytic activity (Fig. S8 and S9, ESI†). The conversion of xanthene reaches >99% within 5 h with a xanthone selectivity of >99% (Fig. 2a). The turnover frequency (TOF)^33^ (3.28 mol kg^−1^ h^−1^; mol of product, per kg of catalyst, per hour) for this reaction over 5 h is significantly higher than those of previously reported reactions conducted with the strong oxidant *tert*-butyl hydroperoxide (^*t*^BuOOH) and MOF catalysts at 343 K: [Fe(BTC)_3_] (TOF = 0.15 mol kg^−1^ h^−1^), [Cu_3_(BTC)_2_] (TOF = 0.12 mol kg^−1^ h^−1^), [Al_2_(BDC)_3_] (TOF = 0.06 mol kg^−1^ h^−1^) (BTC^3−^ = benzene-1,3,5-tricarboxylate; BDC^2−^ = 1,4-benzendicarboxylate), iron citrate (TOF = 0.60 mol kg^−1^ h^−1^), and Fe-exchanged Y zeolite (TOF = 0.006 mol kg^−1^ h^−1^).^33^ The high stability of CNNS/MFM-300(Fe) was confirmed by cycling experiments, with only a very small reduction in yield of xanthone observed over five cycles of photocatalysis (Fig. 2b). The PXRD pattern of CNNS/MFM-300(Fe) after five cycles of photoreaction confirms the retention of crystallinity and structure of MFM-300(Fe) (Fig. S10, ESI†). A leaching test was performed by removing the catalyst from the reaction mixture after 0.5 h. On removal of catalyst a significant drop in reaction rate was observed, thus confirming the key role of CNNS/MFM-300(Fe) and the absence of significant leaching of active sites into solution (Fig. S11, ESI†). The CNNS/MFM-300(Fe) composite also shows high activity and selectivity for the oxidation of indane, thioxanthene and fluorene at room temperature under visible light, demonstrating its general applicability (Table 1). The present system unfortunately shows poor activity toward the oxidation of acyclic benzylic derivatives such as ethylbenzene. Under the same conditions, bulk g-C_3_N_4_ and MFM-300(Fe) can individually catalyse the conversion of xanthene (Fig. 2a), but both show poorer catalytic activity compared with the composite, with values for TOF of 1.34 and 0.49 mol kg^−1^ h^−1^ over 5 h, respectively. Poor dispersion of MOF in g-C_3_N_4_ was observed when the synthesis of the composite was attempted in the absence of CO_2_ or CTAB (Fig. S12 and S13, ESI†), and the catalytic activity of the resultant g-C_3_N_4_-MFM-300(Fe) mixture is much lower than that of the composite prepared in the optimal way (Table 1 and Fig. 2a). This suggests that the high activity of the CNNS/MFM-300(Fe) composite originates from the interface between the two components at and within the heterojunction. When reactions were conducted in dark, negligible catalysis was observed, indicating that the reaction follows a photocatalytic mechanism.

**Table 1:** Photocatalytic conversion of aromatic substrates using CNNS/MFM-300(Fe) composites in the presence of O_2_ at 298 K

Entry	Substrate	Product	Time (h)	Conversion (%)	Selectivity (%)	TOF (mol h^−1^ kg^−1^)
1^a^	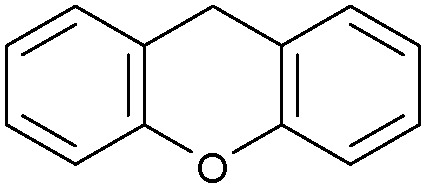	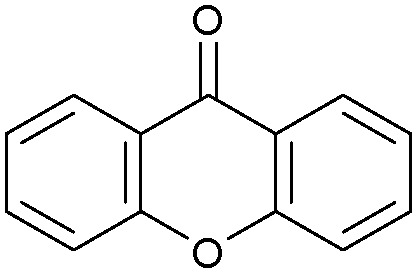	5	>99.0	>99	3.28
2^b^	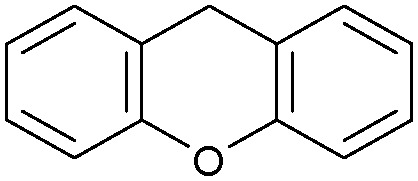	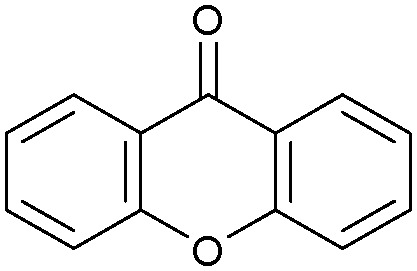	5	47.9	>99	1.59
3^c^	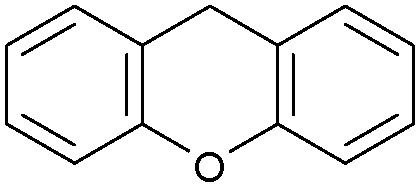	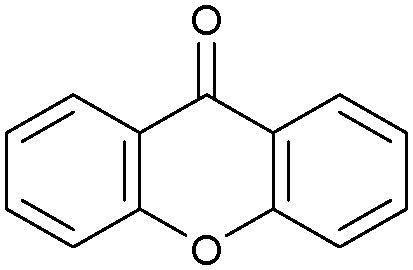	5	59.3	>99	1.95
4	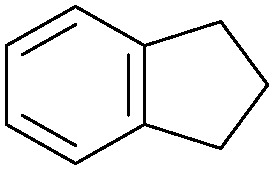	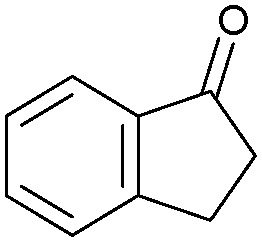	24	91.0	85	0.37
5	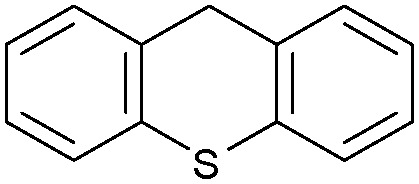	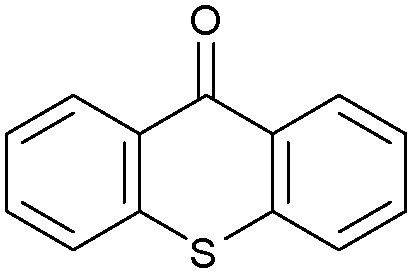	13	96.2	>99	1.12
6	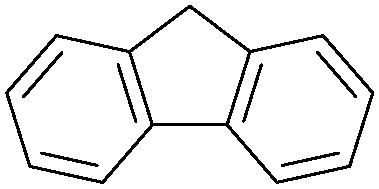	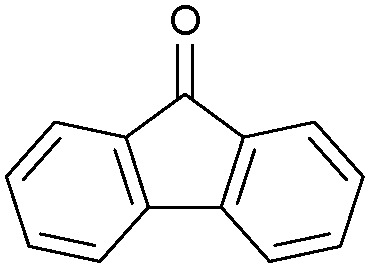	24	87.3	95	0.46
7^d^	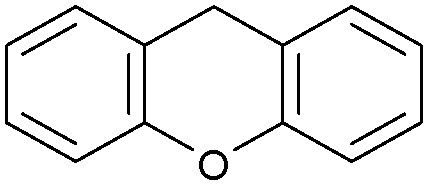	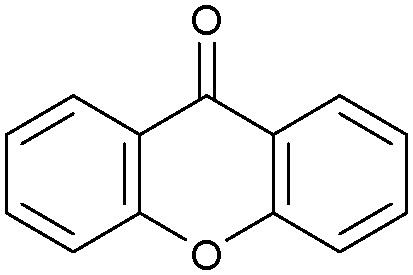	5	52.3	88	1.52
8^e^	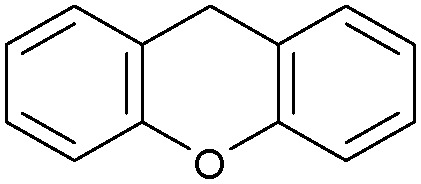	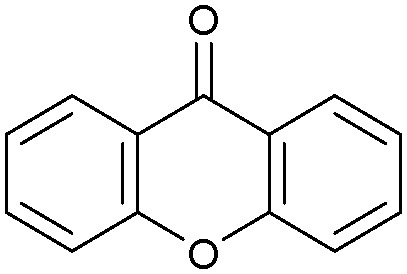	5	14.9	>99	0.49
9^f^	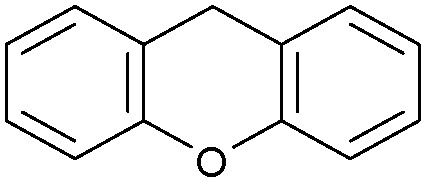	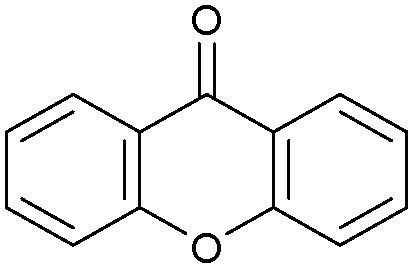	5	41.5	91	1.25
10^g^	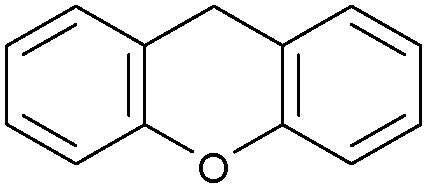	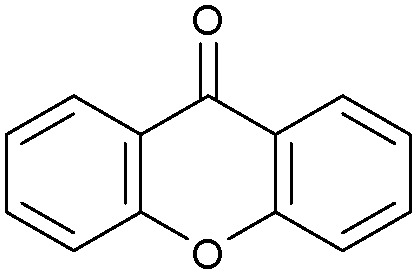	5	2.2	>99	0.07
11^h^	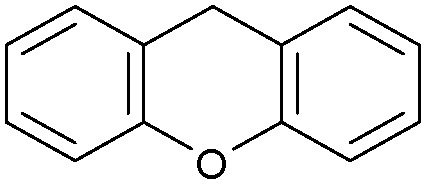	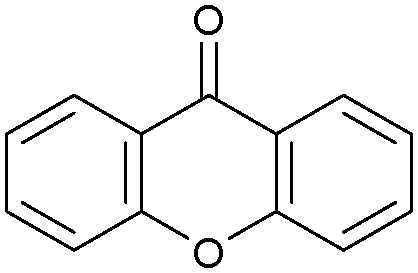	5	0.3	>99	0.01

a Reaction conditions: xanthene (30 mg), CNNS/MFM-300(Fe) (10 mg) (g-C_3_N_4_ : MOF in a wt : wt ratio of 7 : 3), toluene (4 mL), O_2_ (1 bar), *λ* > 400 nm, 298 K.

b Xanthene (30 mg), CNNS/MFM-300(Fe) (10 mg) (g-C_3_N_4_ : MOF in a wt : wt ratio of 7 : 3), MeCN (4 mL), O_2_ (1 bar), *λ* > 400 nm, 298 K.

c Xanthene (30 mg), CNNS/MFM-300(Fe) (10 mg) (g-C_3_N_4_ : MOF in a wt : wt ratio of 7 : 3), trifluorotoluene (4 mL), O_2_ (1 bar), *λ* > 400 nm, 298 K.

d With bulk g-C_3_N_4_ as the photocatalyst (10 mg), xanthene (30 mg), toluene (4 mL), O_2_ (1 bar), *λ* > 400 nm, 298 K.

e With MFM-300(Fe) as the photocatalyst (10 mg), xanthene (30 mg), toluene (4 mL), O_2_ (1 bar), *λ* > 400 nm, 298 K.

f g-C_3_N_4_/MFM-300(Fe) composite synthesised without CO_2_ or CTAB as the photocatalyst (10 mg), xanthene (30 mg), toluene (4 mL), O_2_ (1 bar), *λ* > 400 nm, 298 K.

g Without a catalyst, xanthene (30 mg), toluene (4 mL), O_2_ (1 bar), *λ* > 400 nm, 298 K.

h Without light, CNNS/MFM-300(Fe) (10 mg) (g-C_3_N_4_ : MOF in a wt : wt ratio of 7 : 3), xanthene (30 mg), toluene (4 mL), O_2_ (1 bar), *λ* > 400 nm, 298 K. Other reaction conditions without notes are the same as that used for (a).

### Photocatalysis mechanism study

To rationalise the high catalytic performance of CNNS/MFM-300(Fe), the energy of the optical band gap for MFM-300(Fe), bulk g-C_3_N_4_, CNNS and the CNNS/MFM-300(Fe) composite were determined by electronic spectroscopy to be 3.25, 2.90, 2.99 and 2.97 eV, respectively, using the Tauc-plot method (Fig. S14, ESI†). The flat-band potentials derived from Mott–Schottky plots in the dark are −0.48(3) V and −1.42(4) V *vs.* Ag/AgCl for MFM-300(Fe) and CNNS (Fig. 2c), respectively, suggesting a strong driving force for the photo-excited electrons from CNNS to transfer to MFM-300(Fe). The appropriate band alignment between CNNS and MFM-300(Fe) thus appears to satisfy the thermodynamic requirements for the separation of charge at the interface.

In thermal-catalysed oxidation of hydrocarbons with O_2_, lattice oxygen in the metal oxide catalysts first transfers from the catalyst to the hydrocarbon and the reduced catalyst is subsequently oxidised by O_2_.^34^ We rationalised that the formation of reactive radicals would account for the observed photocatalytic oxidation of benzylic carbons, and we therefore sought to probe the formation of such radicals by EPR spectroscopy *via* spin-trapping experiments. The catalyst CNNS/MFM-300(Fe) suspended in anhydrous toluene gave only very weak EPR spectra in the presence of the spin trap DMPO and molecular O_2_ (1 bar) under dark conditions (Fig. 2d and Fig. S16 and S17, ESI†). On irradiation with visible light, strong EPR spectra were obtained confirming the generation of radical species. The spectra are dominated by the characteristic spectrum of the superoxide adduct DMPO-˙O_2_^−^ (Fig. 2d and Table S1, ESI†). The superoxide anion radicals ˙O_2_^−^ are produced by one-electron reduction of O_2_ with the photogenerated electrons.^35^ In the CNNS/MFM-300(Fe) composite, the redox potential (−1.42 V *vs.* Ag/AgCl) of the conduction band (CB) of CNNS is far more negative than that of MFM-300(Fe) (−0.48 V *vs.* Ag/AgCl) and O_2_/˙O_2_^−^ (−0.33 V *vs.* NHE). Thus, the photogenerated electrons in the conduction band of CNNS can migrate across the heterojunction to the conduction band of MFM-300(Fe), and these accumulated electrons in the conduction band of MFM300(Fe) form a long-lived electron-transfer state, which is favourable for the reduction of O_2_ to ˙O_2_^−^.

The generation of ^•^O_2_^−^ thus relies upon visible light, and the proposed mechanism for the photocatalytic process is summarised in Fig. 2e and f. Upon irradiation, visible light is absorbed by CNNS as evidenced by UV-vis and EPR spectroscopy (Fig. S6 and S17, ESI†) and at the outer layer of the CNNS/MFM-300(Fe) composite electron–hole pairs are generated. The electrons transfer to MFM-300(Fe) where the adsorbed O_2_ is reduced to ˙O_2_^−^ which promotes the oxidation of xanthene to xanthone. The photo-generated holes can effectively migrate to and accumulate at the CNNS, resulting in a spatial separation of electron–hole pairs, enabled by the matching band potentials between MFM-300(Fe) and g-C_3_N_4_.^16,36^

## Conclusions

In summary, a one-pot method has been developed to synthesise CNNS/MFM-300(Fe) composites comprising MFM-300(Fe) grown onto ultra-thin g-C_3_N_4_ nanosheets prepared by exfoliation of bulk g-C_3_N_4_ using scCO_2_. The resultant heterojunctions show excellent photocatalytic activity and product selectivity for oxidation of a range of benzylic C–H groups using O_2_ at room temperature under visible light. Suppression of electron–hole pair recombination at the heterojunction supplies long-lived electrons to MFM-300(Fe) for the formation of superoxide anion radicals, leading to the improved catalytic properties compared with the parent materials. This study will boost the development of new efficient photosynthesis systems using visible light and earth-abundant metal complex catalysts.

## Conflicts of interest

There are no conflicts to declare.

## Supplementary Material

MA-002-D1MA00266J-s001
