# Supplementary material for: Ultra-thin g-C3N4/MFM-300(Fe) heterojunctions for photocatalytic aerobic oxidation of benzylic carbon centers
Source: Mater Adv. 2021 Jul 5;2(15):5144–9. doi: 10.1039/d1ma00266j (PMC8328079; doi:10.1039/d1ma00266j)
Supplement: MA-002-D1MA00266J-s001 [file MA-002-D1MA00266J-s001.pdf]

Electronic Supplementary Material (ESI) for Materials Advances.
This journal is © The Royal Society of Chemistry 2021

Supporting Information

Ultra-thin g-C_3N_4/MFM-300(Fe) heterojunctions for photocatalytic aerobic oxidation of benzylic carbon centers

Chengcheng Liu,[a,b] Tian Luo,[a] Alena M. Sheveleva,[a,c] Xue Han,[a] Xinchen Kang,[a] Sergei Sapchenko,[a] Floriana Tuna,[a,c] Eric J. L. McInnes,[a,c] Buxing Han,[d] Sihai Yang*[a] and Martin Schröder*[a]

a. Department of Chemistry, University of Manchester, Oxford Road, Manchester, M13 9PL (UK).
b. Institute of Molecular Sciences and Engineering, Institute of Frontier and Interdisciplinary Science, Shandong University, Qingdao 266237 (China)
c. Photon Science Institute, University of Manchester, Oxford Road, Manchester, M13 9PL (UK)
d. Beijing National Laboratory for Molecular Sciences, CAS Key Laboratory of Colloid, Interface and Chemical Thermodynamics, Institute of Chemistry, Chinese Academy of Science, Beijing, 100190 (China)

E-mails: Sihai.Yang@manchester.ac.uk; M.Schroder@manchester.ac.uk

Table of Contents

1. Experimental Procedures

1.1 Preparation of g-C_3N_4

1.2 Exfoliation of g-C_3N_4 to CNNS

1.3 Preparation of CNNS/MFM-300(Fe) composites

1.4 Characterisation of materials

1.5 Oxidation of benzylic substrates

1.6 Stability of the catalyst

1.7 Mott-Schottky test

1.8 EPR spectroscopy

2. Results and Discussion

2.1 Exfoliation of bulk g-C_3N_4 to CNNS

2.2 Characterisation of CNNS/MFM-300(Fe)

2.3 Photocatalytic results

2.4 EPR spectroscopy

3. References

1. Experimental Procedures

1.1 Preparation of g-C_3N_4

Bulk g-C_3N_4 was prepared by directly heating urea in air at a rate of 5 K/min to reach a temperature of 823 K and then heated for 4 h.[1]

1.2 Exfoliation of g-C_3N_4 to CNNS

Bulk g-C_3N_4 powder (100 mg) and hexadecyl trimethyl ammonium bromide (CTAB) (380 mg, 1.04 mmol) were dispersed in DMF (5 ml) with 37% HCl aqueous solution (0.25 ml) in a 10ml autoclave. Compressed CO_2 was injected into the autoclave to 75 bar at 293 K and the reaction stirred at 393K for 24 h. On cooling, the compressed CO_2 was released slowly and the as-synthesised material collected via filtration and washed five times with hot DMF and then acetone.

1.3 Preparation of CNNS/MFM-300(Fe) composites

Bulk g-C_3N_4 powder (100 mg), CTAB (380 mg, 1.04 mmol), $FeCl_3\cdot 6H_2O$ (25 mg, 0.09 mmol) and biphenyl-3,3',5,5'-tetracarboxylic acid (H_4L, 30 mg, 0.09 mmol) were dispersed in DMF (5 ml) containing HCl aqueous solution (37%) (0.25 ml) in a 10 ml autoclave. Compressed CO_2 was injected into the autoclave to 75 bar at 293 K and the reaction stirred at 393K for 24 h. On cooling, the compressed CO_2 was released slowly and the as-synthesised material collected via filtration and washed five times with hot DMF and then acetone.

1.4 Characterisation of materials

PXRD data were collected on a Model D/MAX2500, Rigaku X-ray diffractometer using Cu-Kα radiation at a scan rate of 2°/min. FT-IR spectroscopic data were collected on a Thermo Scientific Nicolet iS5 FT-IR spectrometer over the range 400-4000 cm^{-1}, while TGA was conducted at 10°C/min to 800°C under air. Uv-Vis spectra were measured on a Shimadzu UV-2600. In a typical Uv-Vis experiment, the catalyst mixed with $BaSO_4$ powder as support for the measurements. The background for $BaSO_4$ and the reactor has been deducted in all reported spectra.[2] N_2 adsorption-desorption isotherms were measured on a Micromeritics ASAP 2020M system, and the Barrett-Joyner-Halenda method was used for analysis of mesopores. The morphologies of the materials were characterised by SEM on a Quanta 650 and by TEM on a JEM-1011, JEOL. AFM was conducted on a Bruker MM8 Atomic Force Microscope, and GC analysis was conducted on the Agilent Technologies 7890B GC system. The light source used for the photocatalysis was CEL-HXF300, CEAU Light.

1.5 Oxidation of benzylic substrates

All oxidation reactions were carried out in quartz flasks equipped with a O_2 balloon using similar protocols. The oxidation of xanthene is described here in detail. Xanthene (30 mg, 0.165 mmol) and catalyst (10 mg) were loaded into MeCN (4 mL). The air in the flask was replaced by O_2, and the flask was then stirred and irradiated with a 300W Xe lamp equipped with a filter emitting visible light between 400 nm and 1100 nm. The system was kept at 300 K using a water bath. After the reaction, the reaction solution was centrifuged to separate the solid and the upper solution was taken at the targeted reaction time and analysed by GC to determine the yield of product (Figure S18). As all of the substrates and products can be detected in the GC,

the normalisation method was used for the quantification of product yield without the use of internal standard.[3,4] In a typical experiment with xanthene as an example, the GC was equipped with a FID detector with a flow rate for H_2 of 30 mL min^{-1} and a flow rate of air of 400 mL min^{-1}. The equilibration time of the oven was 3 mins and the maximum temperature set to 275 ^{o}C. We calibrated the standard curve using the area of the peak with a known quantity of commercial xanthene and xanthone. The yield of xanthone was obtained from the observed areas of the peaks for xanthene and xanthone in relation to the standard curve.

1.6 Stability of the catalyst

After reaction, the solid catalyst was collected, washed with acetone, dried in air at room temperature for 24 h, and re-used for the next run. Five cycles of repeated tests were conducted. For the leaching test, the reaction mixture at 33% yield after 0.5 h was centrifuged and the supernatant irradiated for 12 h to check for the production of xanthone.

1.7 Mott-Schottky test

Mott-Schottky plots were recorded on a CHI660E workstation (CH Instruments, USA) using a conventional three-electrode system using a 0.5 M Na_2SO_4 aqueous solution. Preparation of the working electrode: 4 mg of bulk g-C_3N_4, CNNS or MFM-300(Fe) were dispersed in a solution of 4 mL ethanol and 10 µL Nafion D-520 to generate homogeneous slurry. 10 µL of the slurry was transferred and coated onto glassy carbon (diameter of 3 mm) and then dried. A Ag/AgCl electrode was employed as the reference electrode, and a platinum plate was used as the counter electrode.

1.8 EPR spectroscopy

Continuous wave (CW) electron paramagnetic resonance (EPR) measurements were carried out at X-band (~9 GHz) at room temperature on Bruker EMX Micro spectrometer with a SHQH-HS-4123D2 resonator. CW EPR spectra of materials before spin-trapping experiments were measured using a 3G modulation amplitude at 9.85 GHz and ~6.3 mW. EPR spectra for the spin-trapping experiments were detected with a modulation amplitude of 1G at 9.85 GHz and ~6.3 mW.

The spin-trap experiments were performed as follows. A solution of DMPO (17.6 mol/L) in dry deoxygenated protonated/deuterated toluene was prepared and stored at -20°C. 2 mg of activated powder materials (treated previously at 10-5 mbar at 120 ^{o}C) were placed in 5mm od quartz tubes in a glove box under N_2 and sealed with silicone stoppers. 3 ml of dry toluene saturated with O_2 (bubbled for 40 min) were injected into the tube, and 2 ml of DMPO solution was injected into the same tube. EPR spectra were recorded before (exp dark) and after (exp light) 10 min of irradiation from a 300W Xe lamp with 400 nm cut-off light filters to remove UV radiation.

Simulation of EPR spectra was performed using EasySpin toolbox (Version 5.2.27) from Matlab. In our simulations, it was assumed that the spectrum consists of superposition of several spectra from different DMPO adducts (components). The evaluation of the relative concentrations of each individual component was made as follows: double integration gave the simulated line shape which was a superposition of all

components calculated (I_{sumsim}). The double integral for each component was then calculated (I_{simi}), and the ratio I_{simi} / I_{sumsim} gave the value of weight of i component (simi) in the resultant simulated spectrum.

2. Results and Discussion

2.1 Exfoliation of bulk g-C_3N_4 to CNNS

Figure S1 shows the PXRD patterns for bulk g-C_3N_4 and the CNNS. Two typical diffraction peaks are observed for bulk g-C_3N_4. The strong peak at 27.37° can be indexed as the diffraction peak of the (002) lattice plane, originating from the characteristic interlayer periodic stacking (d = 0.325 nm) along the *c*-axis in graphitic carbon nitride.[5,6] The weaker diffraction peak at 12.98° corresponds to the (100) lattice plane of g-C_3N_4 with a plane spacing of 0.681 nm, corresponding to the in-plane structural repeating motifs or the heptazine units of the aromatic systems.[5-7] Compared with bulk g-C_3N_4, the graphitic stacking of CNNS is much weakened through the exfoliation by scCO_2 and CTAB as revealed by PXRD in which the peak at 27.37° (002) is of much lower in intensity.[7-9] The intra-layer periodicity (reflex at 2θ =13°) is broadened and drastically weakened due to the geometric confinement, and the in-plane periodicity of the aromatic systems is destroyed on exfoliation.[7,9]

The FT-IR spectra of bulk g-C_3N_4 and CNNS are shown in Figures S1 and S5. The FT-IR spectrum of CNNS is similar to that of bulk g-C_3N_4, suggesting that the structure of CNNS is retained during the exfoliation process. Interestingly, compared with bulk g-C_3N_4, the bands of the composite at 1231, 1305 and 1557 cm^{-1}, which are typical stretching modes of aromatic CN heterocycles, are blue shifted to 1243, 1316 and 1567 cm^{-1}, respectively.[10] This blue shift originates from the decrease of number of layers within 2D materials leading to an increase of in-plane bond energy strengths due to the orthogonal graphitic stacking of CNNS being weakened through the exfoliation.[11] TGA analysis (Figure S1c) shows that the decomposition temperature is lower for CNNS, with these nanosheets more prone to oxidation.

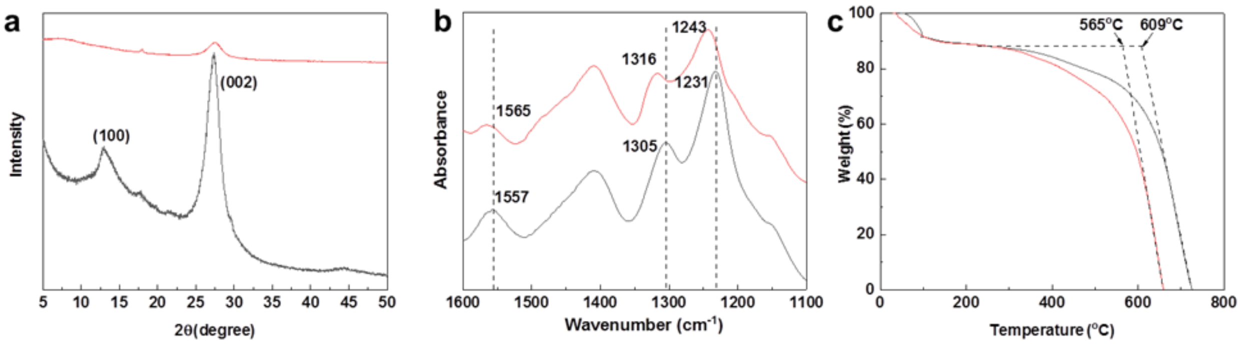

Figure S1: a) PXRD pattern, b) FT-IR spectra and c) TGA of bulk g-C_3N_4 (black) and of CNNS exfoliated using sc-CO_2 and CTAB (red).

UV-visible absorption spectroscopy enabled the calculation of the band gap for bulk g-C_3N_4 and for CNNS as 2.90 eV and 2.99 eV, respectively (Figures S2 and S6).[12] The *n*-type characteristics of bulk g-C_3N_4 and CNNS was confirmed by Mott-Schottky (MS) analysis (Figure S2b) and the flat band potential (fbp) for these two semiconductors are -1.28(3) and -1.42(4) eV, respectively, vs Ag/AgCl.[13] As the g-C_3N_4 and CNNS are *n*-type semiconductors, the fbps are approximately equal to its conduction band potential. The slope of the Mott-Schottky plots for bulk g-C_3N_4 and CNNS were 3.70 x 10^{11} and 0.63 x 10^{11}, respectively, indicating that the carrier density of CNNS is 5.9 times that of bulk g-C_3N_4. This higher carrier density for CNNS facilitates charge transport and is beneficial for the photocatalytic process.[14]

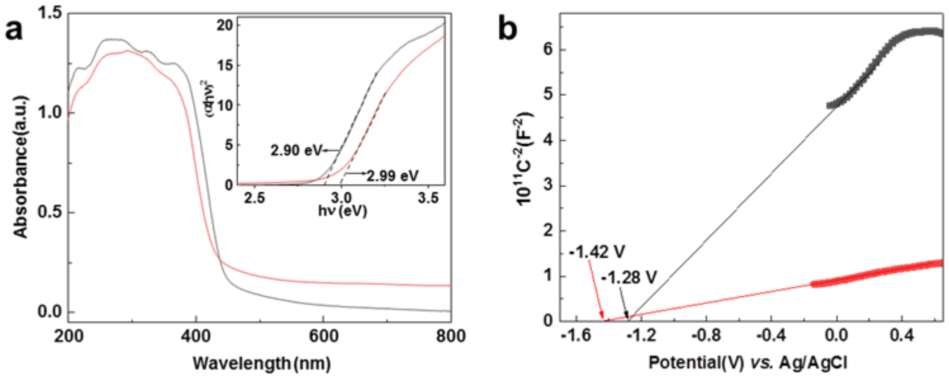

Figure S2 a) Uv-Vis spectra and b) Mott-Schottky plot of bulk g-C_3N_4 (black) and exfoliated CNNS (red). The flat band potential (fbp) of the sample was estimated from the intercept of the X-axis in the linear region of the plot in line with the Mott-Schottky equation.

2.2 Characterisation of CNNS/MFM-300(Fe)

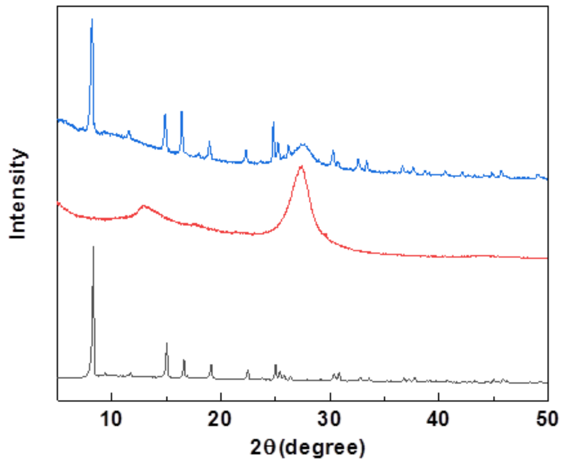

Figure S3: Comparison of PXRD patterns for MFM-300(Fe) (black), bulk g-C_3N_4 (red), and the CNNS/MFM-300(Fe) composite (blue).

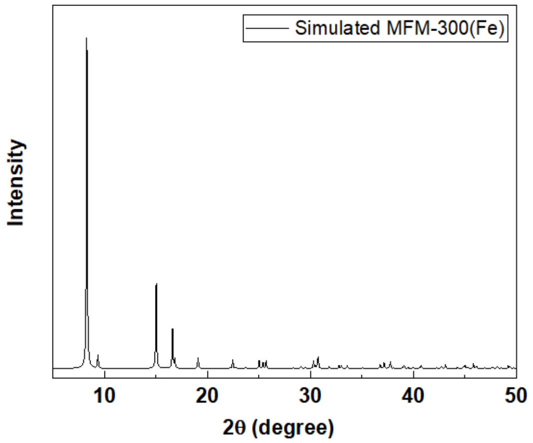

Figure S4: Simulated PXRD pattern of MFM-300(Fe).

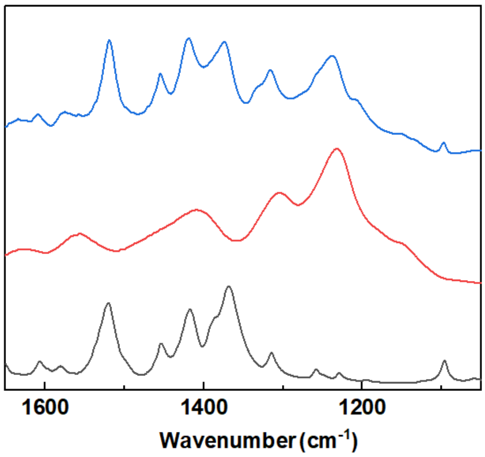

Figure S5: FT-IR spectra of MFM-300(Fe) (black), bulk g-C_3N_4 (red), and the CNNS/MFM-300(Fe) composite (blue).

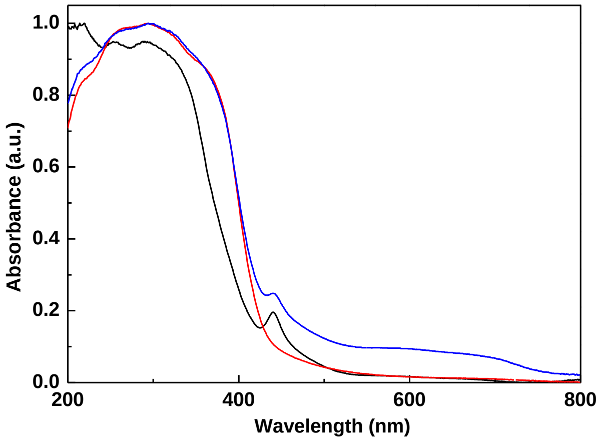

Figure S6: Diffuse reflectance UV-Vis spectra of MFM-300(Fe) (black), bulk g-C_3N_4 (red), and of the CNNS/MFM-300(Fe) composite (blue). The CNNS/MFM-300(Fe) composite shows high background of absorption due to the absorption from exfoliated CNNS sheets, consistent with the comparison of the UV-Vis spectra of bulk g-C_3N_4 and exfoliated CNNS (Figure S2a).

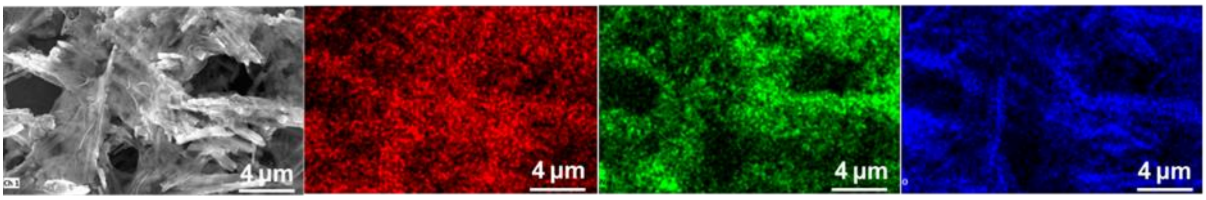

Figure S7: SEM image and elemental mapping of the distribution of Fe (red), N (green), and O (blue) on the surface of the CNNS/MFM-300(Fe) composite.

2.3 Photocatalytic results

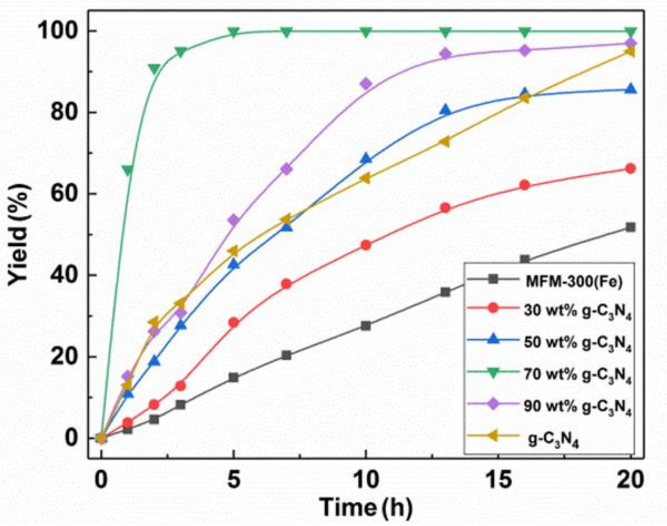

Figure S8: Yield of xanthone using CNNS/MFM-300(Fe) composites of different $g-C_3N_4$ content under visible light at room temperature using a 300 W Xe lamp filtered to emit between 400 nm to 1100 nm.

TGA of the composite shows three steps of weight loss (Figure S9). The weight loss at 351°C is due to the decomposition of MFM-300(Fe). The decomposition temperature for the MFM-300(Fe) synthesized by solvothermal method is about 373°C. The lower decomposition temperature for the MOF is probably due to the smaller particle size of the MOF within the composite. The weight loss of the composite at 430°C is attributed to decomposition of the CNNS. The MFM-300(Fe) content in the composite is about 30wt% calculated from the residue after the calcination.

(Figure S8).

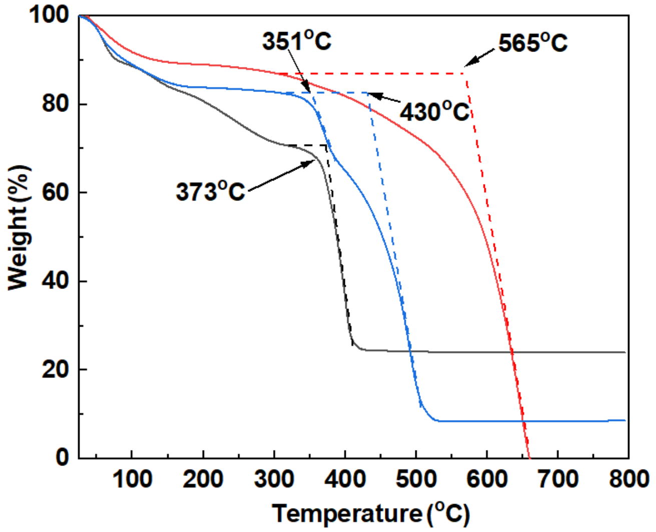

Figure S9 TGA of the MFM-300(Fe) (black), CNNS (red) and the composite of CNNS/MFM-300(Fe) (blue).

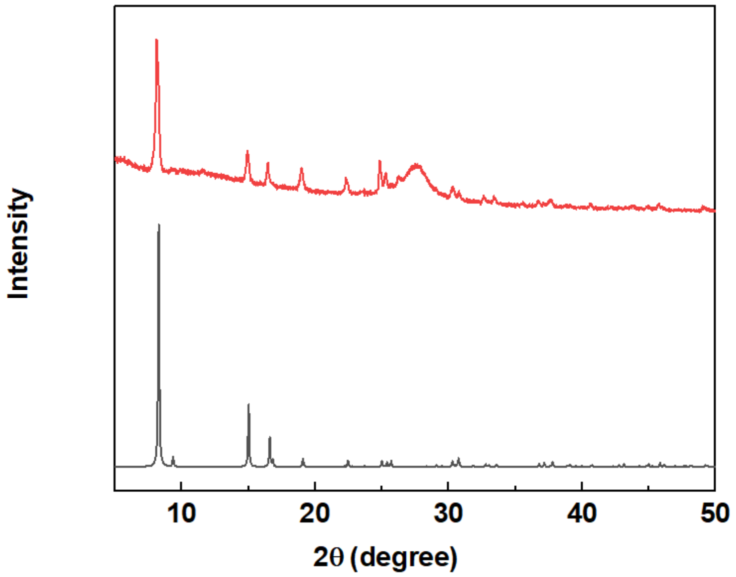

Figure S10: Simulated PXRD patterns of the MFM-300(Fe) (black) and experimental PXRD pattern of the CNNS/MFM-300(Fe) composite after 5 cycles for the oxidation reaction (red).

Leaching tests were performed by removing the catalyst after 0.5 h from the reaction mixture, and the filtrate was stirred for a further 12 h. The reaction progress was monitored by GC as previously described (Figure S11).

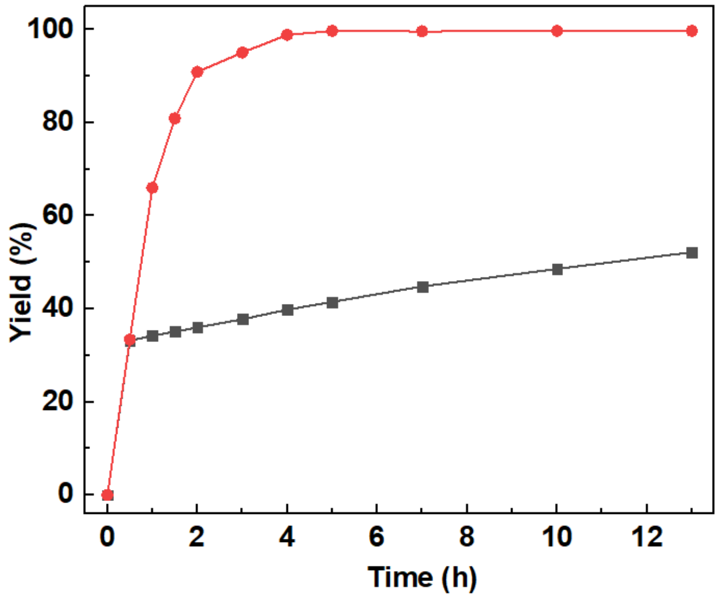

Figure S11: Yield of xanthone over time by oxidation of xanthene with CNNS/MFM-300(Fe) composite as catalyst under visible light at room temperature (red): reaction with catalyst removed after 0.5h (black).

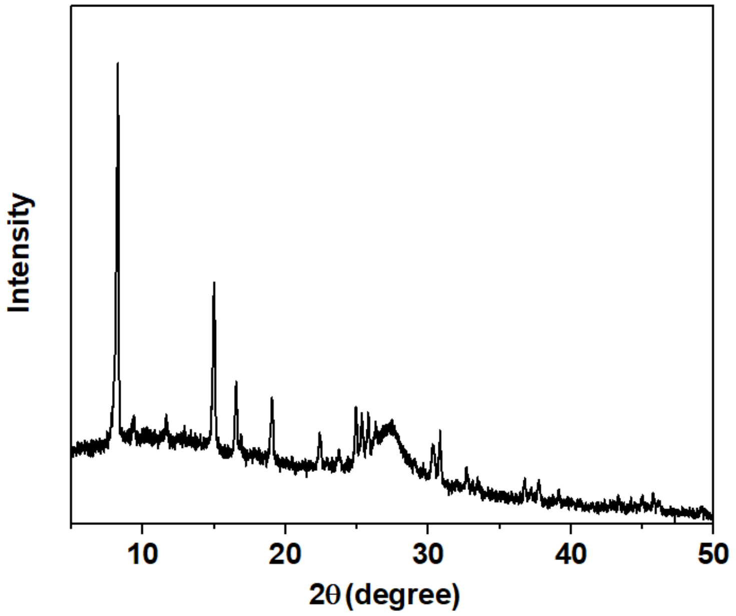

Figure S12: PXRD pattern of the g-C_3N_4/MFM-300(Fe) composite synthesised without CO_2 or CTAB.

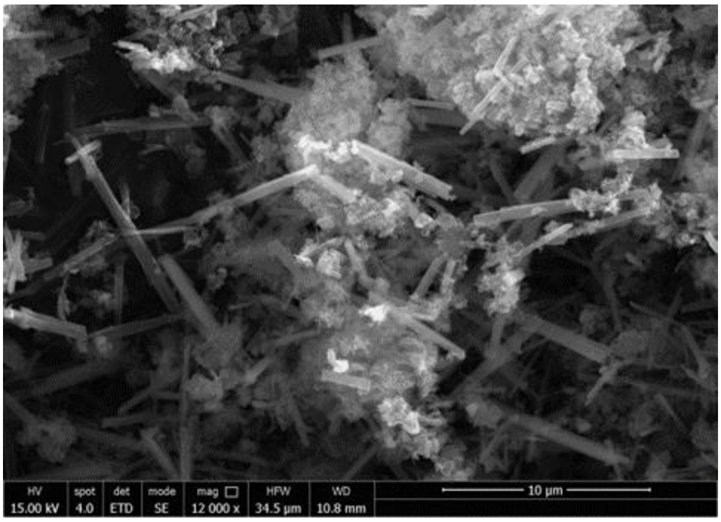

Figure S13: SEM images of the g-C_3N_4/MFM-300(Fe) composite synthesised without CO_2 or CTAB. The g-C_3N_4 remains as multilayer aggregates and the MOF particles are larger than those synthesized in the presence of CO_2 and CTAB.

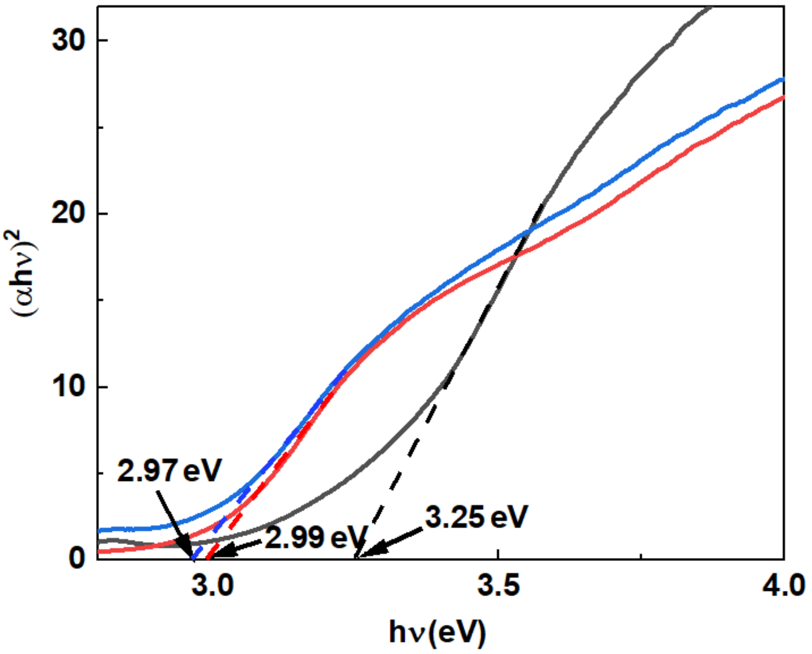

Figure S14: Plot of $(\alpha h\nu)^2$ vs photon energy (hν) of MFM-300(Fe) (black), CNNS (red) and CNNS/MFM-300(Fe) composite (blue) derived from UV/vis spectroscopy.

N_2 adsorption-desorption of g-C_3N_4, MFM-300(Fe) and the composite CNNS/MFM-300(Fe) are shown in Figure S15. The BET (Brunauer-Emmett-Teller) surface area of bulk g-C_3N_4 is 58 $m^2 \cdot g^{-1}$, and that of CNNS/MFM-300(Fe) is much lower (192 $m^2 \cdot g^{-1}$) than MFM-300(Fe) (582 $m^2 \cdot g^{-1}$). This can be ascribed to the coating of CNNS around particles of MOF. Mesopores are formed in the composite material of 7 nm diameter with a mesopore volume about 0.351 $cm^3 \cdot g^{-1}$ derived from Barrett-Joyner-Halenda (BJH) analysis of the pore size

distribution and cumulative volume. This may be attributed to the template effects of the CTAB in the synthesis.

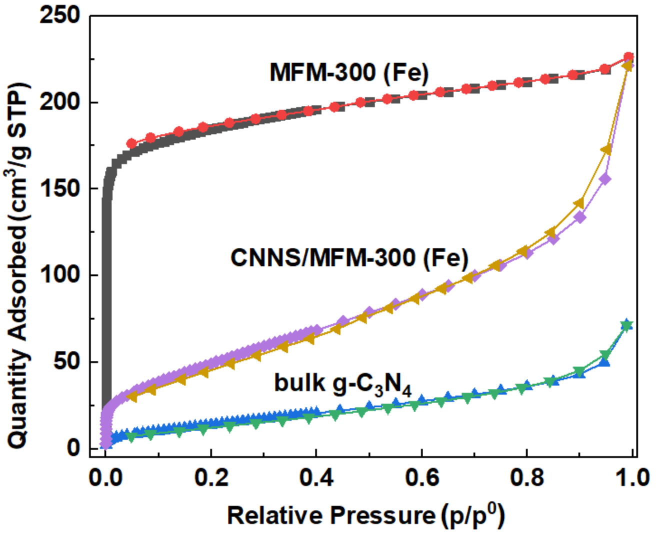

Figure S15: N_2 isotherms (77K) of the MFM-300(Fe), bulk g-C_3N_4 and the composite CNNS/MFM-300(Fe).

2.4 EPR spectroscopy

X-band EPR measurements for all samples before DMPO spin-trap experiments show the presence of a paramagnetic signal (Figure S16). g-C_3N_4 has a weak signal with characteristic g-factor ~2.0036 as reported previously.[15] MFM-300(Fe) has a strong and very broad EPR signal centred on g ≈ 2 due to the paramagnetic Fe^{3+} ions.[16] The composite material CNNS/MFM-300(Fe) has both these features, and a very weak additional peak at g = 4.3 likely due to trace quantities of adventitious Fe^{3+}.[16] These background spectra do not change during the irradiation and spin-trap experiments and hence can be subtracted as baselines.

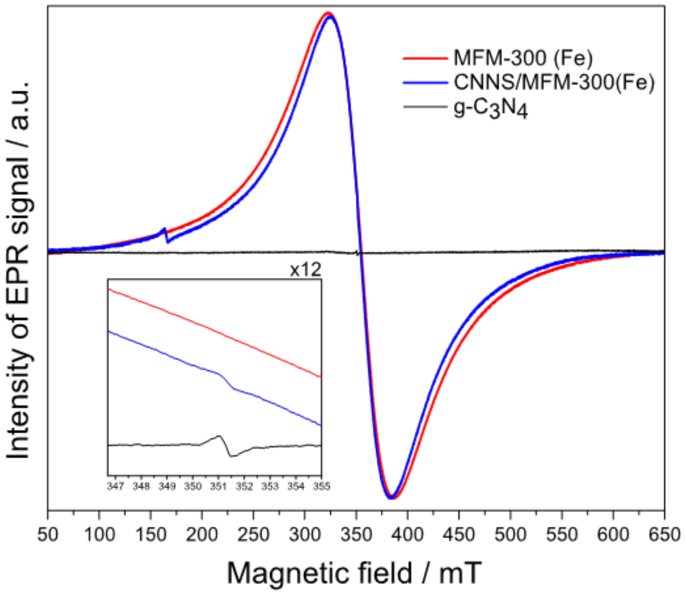

Figure S16: X-band (9.85 GHz) EPR spectra of the MFM-300(Fe), CNNS/MFM-300(Fe) and g-C_3N_4 samples.

DMPO (5,5'-dimethylpyrroline-1-oxide) is a diamagnetic nitrone, widely used for spin-trapping of short-lived free radicals, generating stable nitroxyl radicals (oxidised DMPO) that are readily detected by EPR spectroscopy. The DMPO adducts have a hydrogen at the β- (or 2)-position with respect to the nitroxyl group, which is also the position of radical trapping. The ^{14}N and $^{1}H^{\beta}$ hyperfine coupling are sensitive to the nature of the trapped radical.[17]

For all materials, experiments in deuterated and protonated toluene gave identical spectra (Figure S17), consistent with observation of only spin-trapped C- or O-centred radicals.

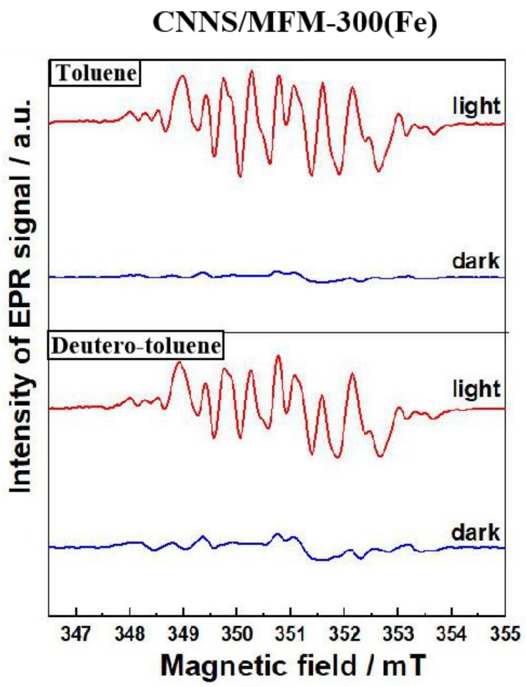

Figure S17: X-band (9.85 GHz) EPR spectra from DMPO spin-trapping experiment with CNNS/MFM-300(Fe) in the presence of oxygen. Upper and lower panels: toluene and deutero-toluene, respectively. For both solvents, data are shown before ("dark") and after ("light") 10 min of visible light irradiation.

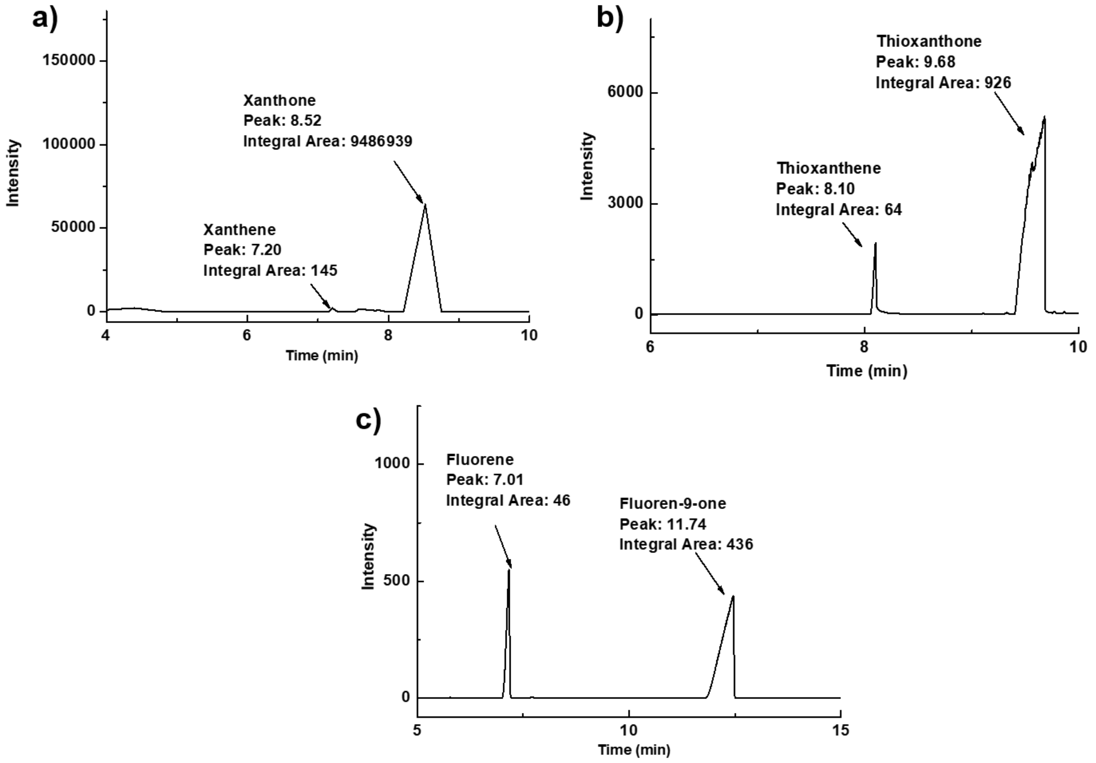

Figure S18: GC plots of the reaction solution after photocatalytic aerobic oxidation of (a) xanthene for 7h, (b) thioxanthene for 13h, and (c) fluorene for 24h.

Table S1: Parameters from simulated EPR spectra presented in Figure 2d of photoloysis of materials in the presence of O_2 in toluene with addition of DMPO.

**CNNS/MFM-300(Fe)**							
Weighting	Adduct	g-value	$A^{14}N$ / G	$A^{\beta}H$ / G	$A^{\gamma}H$ / G	Linewidth/mT	Ref.
0.55	$^{\bullet}O_2^-$	2.0061	12.9	9.6	1.3	0.45	17, 18
0.35	Oxidised DMPO	2.0057	13.9	-	-	0.5	19
0.10	$^{\bullet}CH_2$-C_6H_5	2.0059	14.2	21.5	-	0.3	20

3. References

[1] Y. Zheng, L. H. Lin, B. Wang and X. C. Wang, *Angew. Chem. Int. Ed.,* 2015, **54**, 12868.
[2] X. Shu, J. He, D. Chen, Y. X. Wang, J. Phys. Chem. C, 2008, 112, 4151.
[3] K. Zaitsu, S. Noda, T. Ohara, T. Murata, S. Funatsu, K. Ogata, A. Ishii, A. Iguchi, *Anal. Bioanal. Chem.,* 2019, **411**, 6983.
[4] M. J. Noonan, H. V. Tinnesand, C. D. Buesching, *Bioessays*, 2018, **40**, 1700210.
[5] Y. R. Li, Z. W. Wang, T. Xia, H. X. Ju, K. Zhang, R. Long, Q. Xu, C. M. Wang, L. Song, J. F. Zhu, J. Zhang and Y. J. Xiong, *Adv. Mater.,* 2016, **28**, 6959.
[6] X. C. Wang, K. Maeda, A. Thomas, K. Takanabe, G. Xin, J.M. Carlsson, K. Domen and M. Antonietti, *Nat. Mater.,* 2009, **8**, 76.

[7] S. B. Yang, Y. J. Gong, J. S. Zhang, L. Zhan, L. L. Ma, Z. Y. Fang, R. Vajtai, X. C. Wang and P.M. Ajayan, *Adv. Mater.,* 2013, **25**, 2452.
[8] Y. Wang, X. C. Wang and M. Antonietti, *Angew. Chem. Int. Ed.,* 2012, **51**, 68.
[9] J. S. Zhang, M. W. Zhang, L. H. Lin and X. C. Wang, *Angew. Chem. Int. Ed.,* 2015, **54**, 6395.
[10] J. S. Zhang, M. W. Zhang, R. Q. Sun and X. C. Wang, *Angew. Chem. Int. Ed.,* 2012, **51**, 10145.
[11] G. W. Zhang, S. Y. Huang, A. Chaves, C. Y. Song, V. O. Ozcelik, T. Low and H. G. Yan, *Nat. Commun.,* 2017, **8**, 14071.
[12] P. Niu, L.L. Zhang, G. Liu and H. M. Cheng, *Adv. Funct. Mater.* 2012, **22**, 4763.
[13] L. T. Ma, H. Q. Fan, M. M. Li, H. L. Tian, J. W. Fang and G. Z. Dong, *J. Mater. Chem. A,* 2015, **3**, 22404.
[14] H. X. Zhao, H. T. Yu, X. Quan, S. Chen, H. M. Zhao and H. Wang, *RSC Adv.,* 2014, **4**, 624.
[15] M. Zhang, Y. Y. Duan, H. Z. Jia, F. Wang, L. Wang, Z. Su and C. Y. Wang, *Catal. Sci. Technol.*, 2017, **7**, 452.
[16] R. S. Muralidhara, C. R. Kesavulu, J. L. Rao, R. V. Anavekar and R. P. S. Chakradhar, *J. Phys. Chem. Solids*, 2010, **71**, 1651.
[17] G. R. Buettner, *Free Radic. Biol. Med.*, 1987, **3**, 259.
[18] K. Reszka, P. Bilski and C. F. Chignell, *Free Rad. Res. Comms.*, 1992, **17**, 377.
[19] a) G. D. Feng, P. Cheng, W. F. Yan, M. Boronat, X. Li, J. H. Su, J. Y. Wang, Y. Li, A. Corma, R. R. Xu and J. H. Yu, *Science*, **2016**, **351**, 1188; b) J. M. Fontmorin, R. C. B. Castillo, W. Z. Tang and M. Sillanpaa, *Water Res.* 2016, **99**, 24.
[20] K. J. Reszka and C. F. Chignell, *Photochem. Photobiol.*, 1994, **60**, 442.
